# Clinical, Endocrine, and Reproductive Profiles of Patients with Primary Ovarian Insufficiency at King Faisal Specialist Hospital and Research Centre, Riyadh, Saudi Arabia: A Retrospective Analysis

**DOI:** 10.3390/healthcare14213690

**Published:** 2026-10-31

**Authors:** Hayfa A. AlHefdhi, Mashael Aldeery, Syed Esam Mahmood

**Affiliations:** 1 Department of Family and Community Medicine, College of Medicine, King Khalid University, Abha P.O. Box 641, Saudi Arabia; haifa@kku.edu.sa or dr.hhefdhi@gmail.com; 2 King Faisal Specialist Hospital & Research Centre, Riyadh 11211, Saudi Arabia; maldeery@kfshrc.edu.sa

**Keywords:** primary ovarian insufficiency, premature menopause, turner syndrome, fragile X syndrome

## Abstract

Background: Primary ovarian insufficiency (POI) is a condition causing hypergonadotropic hypogonadism before age 40, leading to menstrual irregularities, infertility, and estrogen deficiency. This study aimed to describe the clinical, endocrine, and reproductive characteristics of patients diagnosed with primary ovarian insufficiency (POI). and describe the distribution of these clinical and biochemical characteristics. Methods: This hospital-based retrospective study was conducted at King Faisal Specialized Hospital and Research Center (KFSHRC) in Riyadh, Saudi Arabia. Medical records of patients diagnosed with POI between 2008 and 2018 were reviewed and analyzed. Results: The onset of POI occurred before 20 years of age in 64.1% of patients. The most common presenting symptoms included infertility (50%), menstrual disturbances (42.2%), delayed puberty (25%), and estrogen deficiency (25%). More than half of the patients were overweight or obese (29.7% each). A family history of POI was reported in 12.5% of cases. Autoimmune diseases were present in 60.9% of patients, while dyslipidemia (25%), and psychiatric disorders (14.1%) were also common. A history of abdominal/pelvic surgery (25%), chemotherapy (28.1%), and radiotherapy (7.8%) was noted. Pelvic ultrasonography revealed that 18.5% of patients had normal findings, while 23/54 (42.6%) had gonadal dysgenesis/agenesis, and 16/54 (29.6%) had uterine abnormalities. Vitamin D deficiency was present in 25/35 (71.4%) of patients, while 3/35 (17.1%) had vitamin D insufficiency, and 3/35 (8.6%) had hypervitaminosis D. Diabetes mellitus was observed in 6/60 (10%) of patients. Autoimmune markers were positive in a subset of patients: anti-thyroid peroxidase antibodies (15/64, 23.4%), anti-thyroglobulin antibodies (12/64, 18.8%), rheumatoid factor (6/64, 9.4%), and other autoimmune markers 3/64 (4.7%). Bone mineral density was low in 23/44 (52.3%) of patients, and 2/44 (4.5%) had delayed bone age. Karyotype analysis identified Turner syndrome in 6/44 (13.6%) of patients, while 38/44 (86.4%) had a normal 46 XX karyotype. Molecular genetic testing for Fragile X syndrome identified one patient with a single gene mutation (1.6%) and one premutation carrier (1.6%). Conclusions: In this cohort, POI was commonly diagnosed before age 20 and was associated with delayed puberty, estrogen deficiency, infertility, and menstrual disturbances. Autoimmune diseases, vitamin D deficiency, and low bone mineral density were also frequently observed. The available data do not support identifying a predominant underlying etiology.

## 1. Introduction

Primary ovarian insufficiency (POI) is characterized by the development of hypergonadotropic hypogonadism before the age of 40 years. Previously referred to as “premature menopause” or “premature ovarian failure”, POI presents with symptoms similar to those of menopause, including menstrual irregularities, estrogen deficiency, and potential infertility [1,2].

Most women with 46, XX spontaneous POI undergo normal puberty and establish regular menstrual cycles before developing the condition [1,3]. The most common presenting symptom is menstrual disturbance, although there is no single characteristic menstrual pattern that predicts its onset [1,2]. While some patients experience sudden amenorrhea, others go through a prolonged phase of oligomenorrhea before the condition is diagnosed [2].

The diagnosis of POI is confirmed in women younger than 40 years who present with irregular menses and follicle-stimulating hormone (FSH) concentrations in the postmenopausal range, as determined by laboratory reference values [4]. Once POI is diagnosed, further investigations are necessary to identify any underlying causes and coexisting conditions. In particular, patients should be evaluated for autoimmune thyroid disease and tested for anti-adrenal antibodies, with additional autoimmune screening guided by clinical indications [4].

The etiology of POI is heterogeneous and largely unknown. Kodaman [5] noted that while POI may occur as an isolated condition, it is often part of a broader clinical syndrome. Despite ongoing research, 90% of POI cases remain idiopathic.

Genetic factors are increasingly recognized as playing a role in POI. Michalakis et al. [6] highlighted the association between rare genetic syndromes and POI. Furthermore, there is emerging evidence linking POI to cardiovascular risk factors, such as lipid abnormalities, blood pressure irregularities, and endothelial dysfunction, which may predispose affected women to cardiovascular disease. These metabolic changes resemble those observed in natural menopause, yet they appear to develop in POI without significant weight gain. The role of adipokines, such as adiponectin and leptin, in these metabolic alterations remains unclear.

In Saudi Arabia, Jaroudi et al. [7] investigated the distribution of human leukocyte antigens (HLA) A, B, Cw, DR, and DQ in POI patients. Their study found no statistically significant difference in HLA antigen distribution between POI patients and controls. However, a positive autoimmune screen was detected in 43.3% of patients, and abnormal thyroid function was identified in 23.3%, reinforcing the link between POI and autoimmune dysfunction.

This study aims to describe the clinical, endocrine, and reproductive characteristics of patients diagnosed with POI and to describe the distribution of their clinical and biochemical features.

## 2. Methodology

This study employed a hospital-based, retrospective data review and analysis design at King Faisal Specialized Hospital and Research Center (KFSHRC) in Riyadh City, Saudi Arabia. The objective was to identify and analyze cases of Primary Ovarian Insufficiency (POI) over a defined period, ensuring a comprehensive review of medical records and patient characteristics.

### 2.1. Study Population and Data Collection

All computerized medical registries at KFSHRC containing records of patients diagnosed with POI between January 2008 and December 2018 were systematically reviewed. The selection process involved identifying patient records using specific diagnostic codes, medical terminologies, and synonyms related to POI. This included alternative terms such as premature ovarian failure, premature menopause, and cases where POI was initially diagnosed as secondary amenorrhea. The inclusion criteria were:

- Female patients diagnosed with POI before the age of 40 and she was less than 40 years old at the time of diagnosis.
- Patients with a confirmed diagnosis based on clinical, biochemical, and hormonal assessments.
- Availability of complete medical records with relevant clinical and demographic data.

Patients with incomplete records, ambiguous diagnoses, or those who did not meet the clinical criteria for POI were excluded from the study. In addition to the above criteria, there was also a need for documented clinical and demographic information, such as age at diagnosis, age at menarche, and age at menopause (if applicable).

This retrospective study reviewed archived medical records of women with an established diagnosis of premature ovarian insufficiency (POI). The diagnosis had been made by the treating physicians before data collection, based on elevated follicle-stimulating hormone (FSH) together with low anti-Müllerian hormone (AMH) and/or low estradiol. FSH, luteinizing hormone (LH), and estradiol were not study outcomes; they were noted in the Methods only to describe the basis of the recorded diagnosis. The archived records did not consistently document these hormone values, the number and timing of measurements, or the menstrual history used to establish the diagnosis, so these data could not be reliably analyzed. The study assessed associated endocrine and autoimmune conditions (hypothyroidism, thyroid autoimmunity, diabetes mellitus, and adrenal insufficiency), as well as vitamin D status and bone mineral density. Reproductive hormone profiles were not evaluated.

The following investigation results were not required to be consistently documented for inclusion in the study: Specific hormone values (e.g., FSH, LH, estradiol levels), the number and timing of hormone measurements, and menstrual history (although clinical information on menstrual status was required). These results could be missing, as they were not considered essential for the study’s inclusion criteria.

A total of 64 eligible patient records meeting the selection criteria were retrieved for further analysis. Data abstraction was conducted manually and cross-verified by two independent researchers to ensure accuracy and minimize bias. The extracted variables included demographic details (age, nationality), clinical presentation (age of onset, symptoms), hormonal profiles (FSH, LH, estradiol levels), genetic testing results, autoimmune associations, and other relevant comorbidities.

At the time of the study, the applicable guideline was the 2016 ESHRE guideline. It defined POI as oligo/amenorrhoea for at least 4 months with FSH > 25 IU/L on two occasions more than 4 weeks apart.

Adherence was assessed by self-report in medical records, without objective measures or validated questionnaires. Additionally, all patients received the same cyclic HRT formulation (Progyluton), the only formulation available at our hospital, limiting generalizability to other regimens.

“Autoimmune disease” referred to any autoimmune condition documented as a diagnosis in the patient’s medical history in the archived records. The most frequent was hypothyroidism (overt or subclinical). Other autoimmune conditions, including type 1 diabetes, psoriasis, polyglandular autoimmune syndrome, autoimmune hepatitis, myasthenia gravis, granulomatosis with polyangiitis, and alopecia areata, were found sporadically.

Genetic testing was recommended for all women with non-iatrogenic primary ovarian insufficiency (POI). Initial investigations included karyotyping, irrespective of age at onset, to identify Turner syndrome or mosaicism, X-chromosome rearrangements, and Y-chromosome material. Patients with Y-chromosome material were referred for specialist counselling regarding gonadectomy because of the associated risk of gonadal tumours. FMR1 premutation testing was also performed in some patients. As this was a retrospective study, genetic testing data were incomplete, and testing was not documented or available for all eligible patients.

BMD was assessed by DXA at the posteroanterior lumbar spine (L1–L4), total hip, and femoral neck. In women with POI, the Z-score is the ISCD-preferred metric for interpretation. A Z-score ≤ −2.0 is described as “BMD below the expected range for age,” and a Z-score > −2.0 as “BMD within the expected range for age.” Because estrogen deficiency is a secondary cause, a Z-score ≤ −2.0 in a woman with POI supports a diagnosis of osteoporosis.

### 2.2. Ethical Considerations

The study was conducted following guidelines of the Declaration of Helsinki and was approved by the Institutional Review Board (IRB) of KFSHRC. Ethical clearance was granted on 2 May 2019, under approval number REF: C380/680/40. The confidentiality of patient data was strictly maintained, and all patient identifiers were removed to ensure privacy and compliance with ethical standards.

### 2.3. Data Entry and Statistical Analysis

The collected data was electronically entered into the Statistical Package for the Social Sciences (SPSS, version 25.0, IBM Corp., Armonk, NY, USA) for analysis. Quality checks were performed to validate the completeness and accuracy of the dataset. Descriptive statistics, including frequencies and percentages, were applied to summarize categorical variables, while means and standard deviations were calculated for continuous variables. Data visualization techniques, such as tables and charts, were utilized to present key findings effectively.

Because of missing data in several variables, all percentages were calculated based on the number of available cases for that specific variable.

By following this rigorous methodology, the study aimed to provide a robust and reliable analysis of POI cases, contributing to a better understanding of its prevalence and associated clinical characteristics in Saudi Arabia.

## 3. Results

Table 1 shows that the age of half the patients (46.9%) was between 30–39 years, while that of 37.5% was <30 years, and that of 15.6% was >40 years. Onset of POI was before 20 years of age among 64.1% of patients, at 20–29 years among 26.6%, while it appeared afterwards among 9.4% of patients. About two thirds of patients (65.6%) were married, while 29.7% were single, and 3.1% were divorced. The majority of patients (92.2%) were nulliparous. The presenting symptoms for POI patients were mainly infertility (50%), menstrual disturbances (42.2%), delayed puberty (25%) and estrogen deficiency (25%). More than half of patients were either overweight or obese (29.7% for each). Family history of POI was positive among 12.5% of patients.

Table 2 shows that POI patients’ main complaints were infertility (50%), menstrual disturbances (42.2%), delayed puberty (25%) and estrogen deficiency (25%). More than half of patients were either overweight or obese (29.7% for each). Most POI patients (60.9%) had associated autoimmune diseases, 25% had dyslipidemia, and 14.1% had associated psychiatric disorders. One-fourth of patients (25%) had past history of abdominal/pelvic surgery, while 28.1% had past history of chemotherapy, and 7.8% had past history of radiotherapy. Table S1 shows that 36 of 64 women had at least one documented autoimmune condition (56.3%).

Table 3 shows that pelvic ultrasonography revealed that 18.5% (10/54) of POI patients were normal, 42.6% (23/54) had gonadal dysgenesis/agenesis, and 29.6% (10/54) had uterine abnormalities. Most POI patients, 25/35 (71.4%), had vitamin D deficiency, while 17.1% (6/35) had vitamin D insufficiency, and 8.6% (3/35) had hypervitaminosis D. Only 10% (6/60) of POI patients were diabetic. About one-fourth of POI patients, 15/64 (23.4%), were positive for anti-thyroid peroxidase antibodies, 18.8% (12/64) for anti-thyroglobulin antibodies, 9.4% (6/64) were positive for rheumatoid factor, and 4.7% (3/64) were positive for markers of other autoimmune diseases. Bone mineral density was low in more than half of POI patients, 23/44 (52.3%), and 2/44 (4.5%) had delayed bone age.

Table 4 shows that karyotype analysis of POI patients revealed that 13.6% (6/44) had Turner syndrome, while the karyotype of 86.4% (38/44) was 46XX. Molecular genetic testing for Fragile X syndrome revealed one patient (1.6%) with a single gene and another patient (1.6%) with premutation carrier.

Table 5 shows that 68.8% of POI patients were compliant toward their hormonal replacement therapy, 81.3% were compliant toward vitamin D supplementation and 82.8% were compliant toward calcium supplementation.

## 4. Discussion

The findings of this study indicate that the onset of premature ovarian insufficiency (POI) predominantly occurs during adolescence, often before the age of 20. Patients typically present with clinical features such as delayed puberty, estrogen deficiency, infertility, and menstrual irregularities. Early-onset POI often results in absent pubertal development, manifesting as delayed puberty or primary amenorrhea, with secondary complications like infertility or secondary amenorrhea emerging later in the disease course [8,9].

Our data revealed that over half of the POI patients were overweight or obese, while 10.9% were underweight. The existing literature suggests that obesity is associated with higher rates of infertility and menstrual irregularities, potentially due to hypothalamic hypogonadism or anovulation [10,11,12]. However, studies show conflicting results regarding BMI’s impact on menopause timing; some report no significant association between BMI and age at menopause among overweight or obese women [13], while others note an earlier menopause in women with low BMI [14]. Notably, Michalakis et al. [6] observed that women with POI tend to be less obese compared to reference groups. Understanding these relationships is important because BMI influences metabolic and reproductive health, which may affect disease progression and management strategies.

Family history plays a significant role, with 12.5% of our patients reporting positive familial patterns, and notable cases of Turner syndrome (13.6%) and Fragile X syndrome (two cases). Genetic abnormalities are recognized as major etiologies in adolescent POI, with studies by Colam et al. [15] and Mishra et al. [16] emphasizing their importance. A positive family history markedly increases the risk, with odds ratios suggesting an 8-fold higher likelihood of premature menopause in women with such history [16]. This underscores the need for early genetic counseling and testing to inform prognosis and guide management.

Genetic counseling may also have implications beyond explaining POI and informing reproductive planning. For example, Abay et al. [17] retrospectively reviewed 48 BRCA1/2 mutation carriers who underwent risk-reducing salpingo-oophorectomy (RRSO). They reported serous tubal intraepithelial carcinoma in one patient, occult ovarian cancer in one, and subsequent peritoneal cancer in another patient during follow-up. These findings highlight the importance of individualized counseling and continued follow-up after RRSO in BRCA1/2 carriers. However, this study addressed cancer risk management in mutation carriers and does not establish BRCA1/2 mutations as an etiology of POI; its relevance to the present cohort is therefore indirect.

Genetic investigations, including linkage analysis and exome sequencing, have identified mutations associated with POI, such as deletions on chromosome 20 [18], and variants linked to menopause timing [19,20,21]. Turner syndrome remains a significant genetic cause, often presenting with short stature, delayed puberty, and infertility, requiring hormone replacement therapy (HRT) to facilitate pubertal development and prevent psychological or skeletal issues [8,22,23]. Recognizing these genetic factors is crucial for early diagnosis and tailored treatment.

The FMR1 gene premutation is another notable genetic factor, with expansions leading to increased POI risk—about 2% prevalence among women with POI—and approximately one-fifth of premutation carriers developing POI [22,23,24,25]. This highlights the importance of genetic screening in at-risk populations to enable early intervention and reproductive planning.

Laboratory assessments revealed that 42.6% of patients had gonadal dysgenesis or agenesis, and 29.6% had uterine abnormalities. Vitamin D deficiency was common, and 10% of patients were diabetic. Autoimmune markers were positive in a significant proportion: anti-thyroid peroxidase antibodies in 23.4%, anti-thyroglobulin antibodies in 18.8%, rheumatoid factor in 9.4%, and other autoimmune markers in 4.7%. Bone mineral density was reduced in over half, with 4.5% exhibiting delayed bone age, highlighting the importance of bone health management to prevent osteoporosis.

Autoimmune diseases, particularly thyroid disorders such as Hashimoto’s thyroiditis, are frequently associated with POI, affecting about one-quarter of patients [5,6,9]. Other autoimmune conditions, including systemic lupus erythematosus and rheumatoid arthritis, have also been linked [9]. The coexistence of autoimmune thyroid disease and POI suggests shared immune dysregulation pathways, emphasizing the need for routine screening and early treatment of autoimmune conditions to preserve ovarian function and overall health.

Vitamin D deficiency is prevalent among POI patients, contributing to low bone mineral density and increased osteoporosis risk [26,27]. Addressing vitamin D deficiency through supplementation may improve bone health outcomes. The association between autoimmune pathology and ovarian decline suggests a multifactorial etiology involving immune dysregulation, which may also influence other systemic health aspects.

Psychiatric comorbidities, especially depression, are more common in women with POI and early menopause, likely due to the psychological impacts of infertility and hormonal deficiencies [28,29,30]. The diagnosis and its implications can adversely affect self-esteem and social roles. Some hypotheses propose shared underlying genetic or neurochemical pathways contributing to both depression and POI [28,31,32,33], highlighting the importance of psychosocial support in comprehensive care.

A history of pelvic surgery, chemotherapy, or radiotherapy was reported in our cohort, with 25% having undergone abdominal or pelvic procedures, 28.1% receiving chemotherapy, and 7.8% exposed to radiotherapy. Pelvic surgeries can impair ovarian blood flow and function [34], while chemoradiation damages primordial follicles and can lead to infertility [35,36,37]. Recognizing these iatrogenic factors is vital for risk assessment and fertility preservation counseling.

Genetic abnormalities remain the predominant cause of adolescent POI, with Turner syndrome being especially common [9]. The link between dyslipidemia and POI has been documented, with studies reporting higher triglyceride levels and lower HDL cholesterol among affected women, raising concerns about increased cardiovascular risks [38,39].

Regarding management, approximately two-thirds of patients adhered to hormone replacement therapy (HRT), with high compliance toward vitamin D and calcium supplementation. HRT is recommended to alleviate vasomotor symptoms, support bone health, and mitigate cardiovascular risks [40,41]. Regular follow-up is essential to monitor side effects, adherence, and treatment efficacy, with patient involvement in decision-making improving compliance.

A significant number of patients in this study did not take or discontinued hormone replacement therapy (HRT). This could be due to lack of interest or concerns about breast cancer. Importantly, existing evidence shows that HRT does not increase breast cancer risk beyond the levels seen in women with normal menstruation [42]. Another study examined the long-term follow-up of POI patients, highlighting poor compliance with HRT despite its crucial role in preventing bone demineralization [43].

Given the chronic nature of POI and its potential for serious long-term health consequences, prospective studies are essential to better understand the long-term health impacts of HRT in these patients. Such research would clarify its role in preventing osteoporotic fractures and supporting cardiovascular health, ultimately guiding more effective, individualized management strategies.

This study employed a retrospective design and has several limitations. First, substantial missing data and incomplete medical records—particularly for karyotype, vitamin D, and bone mineral density—meant that prevalence estimates for these variables could be calculated only among participants with available results. Because testing may have been guided by clinical indications, missing data may not be random and could have biased these estimates. Accordingly, the reported proportions may not represent the full cohort and should be interpreted cautiously, especially when denominators are small. Second, the small sample from a single tertiary referral center may introduce referral bias, potentially overrepresenting complex, genetic, or iatrogenic cases of POI compared with the general population. Finally, because the study used descriptive analyses and did not test associations, the findings do not support conclusions about relationships among the variables, including BMI. Finally, because the study used descriptive analyses and did not test associations, the findings do not support conclusions about relationships among the variables, including BMI; the absence of a control group further limits the interpretation of these findings. Consequently, some results could not be fully obtained or analyzed, potentially affecting the comprehensiveness of the findings.

In this tertiary-center Saudi cohort, many patients with POI were diagnosed before age 20. Delayed puberty, estrogen deficiency, and menstrual disturbances were commonly documented clinical features. Autoimmune conditions, vitamin D deficiency, and chromosomal abnormalities were also observed. However, given the small, referral-based cohort and the potential overlap among genetic, autoimmune, and treatment-related factors—including chemotherapy, radiotherapy, and prior surgery—we cannot reliably identify a single predominant etiology or generalize these findings to all patients with POI. Referral bias may have contributed to the clinical and etiological profile observed. Adherence to hormone replacement therapy (HRT) was suboptimal, underscoring the importance of individualized care and patient education. Larger, multicenter studies with systematic etiological classification are needed.

Finally, because of the retrospective design, diagnostic criteria may not have been applied consistently across the cohort, and we could not verify that all cases met the applicable 2016 ESHRE criteria: oligo/amenorrhoea for at least 4 months and FSH > 25 IU/L on two measurements more than 4 weeks apart. The updated 2024 ESHRE/ASRM/CRE/IMS guideline requires a single elevated FSH measurement, with repeat testing when diagnostic uncertainty remains. Differences in diagnostic criteria and documentation over time may therefore have affected case ascertainment and limit comparability with studies using current criteria. We acknowledge that self-reported adherence may overestimate actual use, and is a limitation of our study.

## 5. Conclusions

In this retrospective cohort from a tertiary referral center in Riyadh, POI was frequently identified before age 20 and was commonly associated with delayed puberty, menstrual disturbances, estrogen deficiency, and infertility. Autoimmune conditions, vitamin D deficiency, and low bone mineral density were also observed, while karyotype findings included Turner syndrome in a subset of patients. These results support comprehensive assessment of patients with POI, including evaluation of possible genetic and autoimmune conditions, bone health, and metabolic comorbidities, alongside individualized counseling and management. HRT adherence was suboptimal in this cohort, highlighting the value of clear patient education and ongoing follow-up. Because the study was retrospective, single-center, and affected by missing data and incomplete diagnostic documentation, its findings should be interpreted cautiously and should not be considered representative of all patients with POI in Saudi Arabia. Larger, multicenter studies using systematic diagnostic and etiological assessment are needed.

## Figures and Tables

**Table 1:** Personal characteristics of patients with primary ovarian insufficiency (N = 64).

Personal Characteristics	No. (n)	% (n/N)
Age at data collection		
<30 years	24	37.5
30–39 years	30	46.9
≥40 years	10	15.6
Age at onset of primary ovarian insufficiency		
<20 years	41	64.1
20–29 years	17	26.6
30–39 years	6	9.4
Marital status		
Married	42	65.6
Single	19	29.7
Divorced	2	3.1
Widow	1	1.6
Parity		
0	59	92.2
≥1	5	7.8
Body mass index		
Underweight	7	10.9
Normal	19	29.7
Overweight	19	29.7
Obese	19	29.7
Family history of primary ovarian insufficiency	8	12.5

**Table 2:** Clinical characteristics of patients with primary ovarian insufficiency (N = 64).

Clinical Characteristics	No. (n)	% (n/N)
Main presenting complaints		
Delayed puberty	16	25.0
Menstrual cycle disturbances	27	42.2
Estrogen deficiency	16	25.0
Infertility	32	50.0
Associated comorbidity		
Autoimmune diseases	39	60.9
Dyslipidemia	16	25.0
Low bone mineral density		
Psychiatric disorders	9	14.1
Past history of abdominal/pelvic surgery	16	25.0
Past history of chemotherapy	18	28.1
Past history of radiotherapy	5	7.8

**Table 3:** Results of laboratory investigations for patients with primary ovarian insufficiency.

Laboratory Investigations	No. (n)	(n/N) %	95% CI
Pelvic ultrasonography ^(1)^ (N = 54)			
Normal	10	18.5	10.4–30.8%
Gonadal dysgenesis/agenesis	23	42.6	30.3–55.9%
Hemorrhagic ovarian cyst	2	3.7	1.0–12.5%
Mullerian anomaly	3	5.6	1.9–15.1%
Uterine abnormalities	16	29.6	19.2–42.8%
Serum vitamin D assessment ^(2)^ (N = 35)			
Normal	1	2.9	0.5–14.5%
Deficiency	25	71.4	55.0–83.0%
Insufficiency	6	17.1	8.1–32.7%
Hypervitaminosis D	3	8.6	3.0–22.4%
HbA1c ^(3)^ (N = 60)			
Normal (<5.6%)	42	70.0	57.5–80.1%
Prediabetic (5.7–6.4%)	12	20.0	11.8–31.8%
Diabetic (>6.5%)	6	10.0	4.7–20.2%
Positive Autoimmune markers (N = 64)			
Anti-thyroid peroxidase	15	23.4	14.7–35.1%
Anti-thyroglobulin	12	18.8	11.1–29.9%
Rheumatoid factor	6	9.4	4.4–19.0%
Others	3	4.7	1.6–12.9%
Bone mineral density ^(4)^ (N = 44)			
Normal	17	38.6	25.7–53.3%
Low	23	52.3	38.0–66.3%
Delayed bone age	2	4.5	1.3–15.1%
Fracture	2	4.5	1.3–15.1%

^(1)^ Results of pelvic ultrasonography are missing for 10 cases. ^(2)^ Serum vitamin D was not assessed for 29 cases. ^(3)^ Results of HbA1c are missing for 4 cases. ^(4)^ Results of bone mineral density are missing for 20 cases.

**Table 4:** Results of karyotype analysis for patients with primary ovarian insufficiency.

Karyotype Analysis	No.	% (n/N)
Karyotype (N = 44)		
Turner syndrome	6	13.6
46XX	38	86.4
Fragile X syndrome (N = 64)		
Single gene	1	1.6
Premutation	1	1.6

The karyotypes of 20 cases are missing.

**Table 5:** Compliance of patients with primary ovarian insufficiency toward received treatment (N = 64).

Received Treatment	No.	% (n/N)
Hormonal replacement therapy		
Yes	44	68.8
No	16	25.0
Discontinued	4	6.3
Vitamin D supplementation		
Yes	52	81.3
No	9	14.1
Discontinued	3	4.7
Calcium supplementation		
Yes	53	82.8
No	10	15.6
Discontinued	1	1.6

## Data Availability

The original contributions presented in this study are included in the article. Further inquiries can be directed to the corresponding author.

## Supplementary Materials

The following supporting information can be downloaded at: https://www.mdpi.com/article/10.3390/healthcare14213690/s1, Table S1: Autoimmune conditions in women with premature ovarian insufficiency (n = 64).

## Abbreviations

The following abbreviations are used in this manuscript:

POI	Primary ovarian insufficiency
HRT	Hormone replacement therapy
KFSHRC	King Faisal Specialized Hospital and Research Center
