# Supplementary material for: Clinical, Endocrine, and Reproductive Profiles of Patients with Primary Ovarian Insufficiency at King Faisal Specialist Hospital and Research Centre, Riyadh, Saudi Arabia: A Retrospective Analysis
Source: Healthcare (Basel). 2026 Oct 31;14(21):3690. doi: 10.3390/healthcare14213690 (PMC13762564; doi:10.3390/healthcare14213690)
Supplement: Supplementary file 1 [file healthcare-14-03690-s001.zip › healthcare-4581556-supplementary.pdf]

Supplementary Table S1. Autoimmune conditions in women with premature ovarian insufficiency (n = 64)

Autoimmune condition	n	%
**Any autoimmune condition**	**36**	**56.3**
**Autoimmune thyroid disease**	**30**	**46.9**
Overt hypothyroidism	22	34.4
Subclinical hypothyroidism	8	12.5
Hyperthyroidism (later hypothyroid)	1	1.6
Polyglandular autoimmune syndrome	2	3.1
Psoriasis	2	3.1
Type 1 diabetes	1	1.6
Adrenal insufficiency	1	1.6
Hypoparathyroidism	1	1.6
Autoimmune hepatitis	1	1.6
Myasthenia gravis	1	1.6
Granulomatosis with polyangiitis	1	1.6
Alopecia areata	1	1.6

Among the 64 women, 36 (56.3%) had at least one documented autoimmune condition. Autoimmune thyroid disease was the most common, reported in 30 women (46.9%), including 22 (34.4%) with overt hypothyroidism and 8 (12.5%) with subclinical hypothyroidism. One woman (1.6%) had a history of hyperthyroidism followed by hypothyroidism. Other recorded conditions included polyglandular autoimmune syndrome and psoriasis (2 women each; 3.1% each), and type 1 diabetes, adrenal insufficiency, hypoparathyroidism, autoimmune hepatitis, myasthenia gravis, granulomatosis with polyangiitis, and alopecia areata (1 woman each; 1.6% each). Conditions could overlap.
